# *Sedum xinchangense*, a new species of Crassulaceae from Zhejiang, East China

**DOI:** 10.3897/phytokeys.272.188016

**Published:** 2026-04-07

**Authors:** Zhi-Dan Wang, Xiao-Feng Jin, Yi-Fei Lu

**Affiliations:** 1 School of Forestry and Biotechnology, Zhejiang A&F University, Hangzhou, 311300, Zhejiang, China Zhejiang A&F University Hangzhou China

**Keywords:** Morphology, nr-ITS, phylogeny, sect. *Sedum*, taxonomy

## Abstract

*Sedum xinchangense* (Crassulaceae), a new species from Zhejiang, eastern China, is described with illustrations. The new species is morphologically similar to *S. lungtsuanense* and *S. stellariifolium*, but can be distinguished by a combination of morphological characters, including the presence of stolons, leaf and sepal shape, indumentum type, flower number, and leaf and seed size. Phylogenetic analyses also confirmed its distinct species status, placing it within the *Acre* clade of subfamily Sempervivoideae. Based on both morphological and molecular evidence, we assign the new species to sect. *Sedum*.

## Introduction

Crassulaceae, a family of succulent plants comprising 1410 species across 34 genera, represents the largest family within the order Saxifragales (Van Ham and Hart 1998; Mort et al. 2001; APG IV 2016). *Sedum* L. is the most species-rich genus within the family and is widely distributed, comprising approximately 470 species (Thiede and Eggli 2007). The genus is mainly distributed across the Northern Hemisphere, with a limited number of species native to Africa and South America (Fu and Ohba 2001). Major centers of diversity for *Sedum* include the Mediterranean region, Central America, the Himalayas, and East Asia (Stephenson 1994).

Based on the integration of morphological and molecular data, Thiede and Eggli (2007) divided Crassulaceae into three subfamilies, viz. Crassuloideae, Kalanchoideae, and Sempervivoideae, a classification widely adopted in subsequent studies (Gontcharova and Goncharov 2009; Messerschmid et al. 2020; Ding et al. 2022; Liu et al. 2023; Han et al. 2024). Among these, Sempervivoideae is the most species-rich and morphologically complex. Recent systematic studies recognize it as comprising five clades (*Telephium*, *Sempervivum*, *Aeonium*, *Leucosedum*, and *Acre* clades) (Chang et al. 2021; Liu et al. 2023; Han et al. 2024) or six major clades (when *Sempervivum* is further split into the *Petrosedum* and *Sempervivum*/*Jovibarba* lineages) (Messerschmid et al. 2020). A key finding emerging from these modern phylogenetic analyses is that the genus *Sedum*, as traditionally circumscribed, is highly polyphyletic (or paraphyletic), with its species scattered across multiple clades within Sempervivoideae (Carrillo-Reyes et al. 2009; Mort et al. 2010; Messerschmid et al. 2020).

In China, *Sedum* is morphologically divided into three sections: sects. *Sedum*, *Oreades* (Fröd.) K.T.Fu, and *Filipes* (Fröd.) S.H.Fu (Fu and Ohba 2001), encompassing 121 species, of which 91 are endemics. Section *Sedum* is distinguished from the others by its carpels and follicles, which are adaxially gibbous. The primary morphological distinctions between sect. *Oreades* and sect. *Filipes* are the presence or absence of a leaf base spur and petal coloration.

The taxonomic study of *Sedum* in Zhejiang began in the late 19^th^ century, with the collection and description of *S. polytrichoides* Hemsl. from Ningbo (Forbes and Hemsley 1887). Subsequent work by Migo, who conducted extensive field collections in eastern China, led to the description of additional species, including *S. emarginatum* Migo from Lingyin of Hangzhou City (Migo 1937). Following the mid-20^th^ century, intensified fieldwork and taxonomic studies resulted in the publication of several new *Sedum* species from Zhejiang: *S. lungtsuanense* S.H.Fu (Fu 1965), *S. angustifolium* Z.B.Hu & X.L.Huang (Hu et al. 1981), *S. hangzhouense* K.T.Fu & G.Y.Rao, and another new subspecies, *S. tosaense* subsp. *sinense* K.T.Fu & G.Y.Rao (Fu and Rao 1988), and *S. ecalcaratum* H.J.Wang & P.S.Hsu (Hsu 1991). During the preparation of the “Flora of Zhejiang” treatment of the Crassulaceae, Ho (1989) described two further new species, *S. jiulungshanense* Y.C.Ho and *S. tianmushanense* Y.C.Ho & F.Chai. Our own taxonomic investigations have contributed to the new species *S. hoi* X.F.Jin & B.Y.Ding (Wang et al. 2005), *S. kuntsunianum* X.F.Jin, S.H.Jin & B.Y.Ding (Jin et al. 2013), as well as a new variety *S. drymarioides* var. *saxifragiforme* X.F.Jin & H.W.Zhang (Jin et al. 2010). We also synonymised *S. angustifolium* with *S. sarmentosum* Bunge, and *S. ecalcaratum* with *S. kiangnanense* D.Q.Wang & Z.F.Wu, respectively (Jin et al. 2010). In recent years, several new *Sedum* species from Zhejiang have been described, including *S. plumbizincicola* X.H.Guo & S.B.Zhou ex L.H.Wu, *S. xunvense* Y.L.Xu & P.Li, *S. simingshanense* Y.L.Xu, and *S. orientalichinense* Q.Fan & P.Li (Wu et al. 2013; Chai et al. 2024; Dai et al. 2025; She et al. 2025).

During a botanical exploration of *Sedum* in eastern to central China, we collected a plant closely resembling *S. lungtsuanense* and *S. stellariifolium* on Mt. Shijiufeng in Xinchang County of Zhejiang, eastern China. It grows on cliffs and bears basal stolons; the stems, leaves, and bracts are densely glandular. The leaf blade is spatulate, and the sepals are obovate to broadly obovate. Based on detailed morphological comparison and phylogenetic analysis, we here confirm it as a new species and provide its formal description below.

## Materials and methods

### Observation and comparison of morphological characters

The new species was collected from Mt. Shijiufeng (Xinchang County, Zhejiang Province, China) and cultivated at Zhejiang A&F University until flowering. Voucher specimens were deposited in ZJFC. Morphological observations were conducted on both living plants and specimens of the new species. Given its morphological resemblance to *S. lungtsuanense* and *S. stellariifolium*, specimens of these two species were examined from HHBG, NAS, PE, and ZM (accessed via https://www.cvh.ac.cn/). Detailed morphological comparisons were subsequently made among the three taxa to identify diagnostic characters, based on our specimen measurements and data from Fu (1965), Fu and Fu (1984), Fu and Ohba (2001), and Jin et al. (2008). In addition, pollen grains and seeds of the new species were imaged using a SU8010 scanning electron microscope (SEM) (Hitachi High-Tech, Japan). A total of 30 pollen grains and 20 seeds were measured from the SEM images using ImageJ (Collins 2007).

### Taxon sampling for phylogenetic analyses

A total of 111 samples representing 102 species (including infraspecific taxa) were included in the phylogenetic analyses. These samples span six major clades of Sempervivoideae, with members of Kalanchoideae serving as the outgroups (Messerschmid et al. 2020). The two samples of the new species were newly generated for this study, and the corresponding raw data have been submitted to the China National Center for Bioinformation (https://ngdc.cncb.ac.cn/gsa/) under the BioProject accession number PRJCA056148. 109 ITS sequences and three plastid genes (46 *matK*, 37 *rps16*, and 64 *trnL-F*) of Crassulaceae were downloaded from NCBI. The species names and GenBank accession numbers are listed in Suppl. material 1: table SS1.

### DNA extraction and sequencing

The fresh leaves of new species were collected and preserved in silica gel. Total genomic DNA was extracted using a modified CTAB method (Doyle and Doyle 1987). To perform deep genome skimming, purified DNA was fragmented by ultrasonication (Covaris E220, Covaris, Brighton, UK) and size-selected for fragments of 300–500 bp using magnetic beads. Paired-end libraries were prepared according to the manufacturer’s instructions (China National GenBank, Shenzhen, China) and sequenced on a DNBSEQ-T7 platform to generate 150 bp paired-end reads. Using the published genome size of *S. album* (approximately 610 Mbp; Wai et al. 2019) as reference, two samples of the new species were sequenced to approximate depths of 14 × and 49 ×, yielding 8.53 Gb and 30.02 Gb of raw data, respectively.

### Phylogenetic analyses

Raw sequencing data were quality-controlled and filtered using SOAPnuke v.1.5.6 (Chen et al. 2018) with parameters “-n 0.1 -q 0.5 -l 12”. The resulting clean reads were further assessed with FastQC v.0.12.0 (Andrews 2019) to verify data quality. The downloaded sequences were used as references to extract the ITS sequences and plastid genes from two newly sequenced samples using GeneMiner v.2.1 (Xie et al. 2024). Sequences were aligned using the online L-INS-i algorithm in MAFFT (https://mafft.cbrc.jp/alignment/server/), and the resulting alignments were trimmed and manually edited in MEGA v.7 (Kumar et al. 2016). The best nucleotide substitution model was determined for each gene region under the Bayesian Information Criterion (BIC) in jModelTest v.2.1.10 (Guindon and Gascuel 2003; Darriba et al. 2012). Finally, *matK*, *rps16*, and *trnL-F* regions were concatenated in PhyloSuite v.1.2.3 (Zhang et al. 2020). Phylogenetic trees were inferred using both Maximum likelihood (ML) and Bayesian Inference (BI) approaches. The ML tree was constructed using RAxML-HPC BlackBox v.8.2.12 (Stamatakis 2014) in the CIPRES Science Gateway (https://www.phylo.org/) with 1000 bootstrap replicates. For BI tree, the analysis was performed in MrBayes v.3.2.7a (Ronquist et al. 2012) under the selected substitution model. Two independent runs of four chains each were conducted for 30 million generations, sampling trees every 1000 generations. The first 25% of sampled trees were discarded as burn-in, and the remaining trees were used to generate a majority-rule consensus tree. The phylogenetic trees were visualized in tvBOT (Xie et al. 2023).

## Results

### Comparison of morphological characters

Based on the examination of specimens, review of relevant literature, and field observations, we compared the morphological characters of the new species with those of *S. lungtsuanense* and *S. stellariifolium* (Table 1). The new species differs from both in its basal stolons (absent in the latter two), sepal shape, and larger leaves and seeds. It can be further distinguished from *S. lungtsuanense* by its densely glandular indumentum and fewer-flowered cymes (3–14 vs. 12–23), and from *S. stellariifolium* by its spatulate leaf blade, rarely obovate or broadly obovate (vs. triangular to broadly triangular-ovate). Quantitative measurements confirm its distinctiveness: leaves 5–20 × 3.5–13 mm (vs. 7–9 × 2.2–3 mm in *S. lungtsuanense* and 7–15 × 7–9 mm in *S. stellariifolium*), seeds ca. 0.56 × 0.32 mm (vs. ca. 0.29 × 0.15 mm in *S. lungtsuanense* and ca. 0.38 × 0.24 mm in *S. stellariifolium*).

**Table 1.** Morphological comparison among *Sedum xinchangense*, *S. lungtsuanense*, and *S. stellariifolium*.

Species\ Characters	*S. xinchangense*	*S. lungtsuanense*	*S. stellariifolium*
Life form	perennial	annual	annual or biennial
Habitat	on cliffs	under hillside forest canopies or on shaded, moist rocks	soil or rock crevices in valleys or on slopes
Stolon	present	absent	absent
Fertile stem	densely glandular	sparsely pubescent	densely glandular
Inflorescence	cyme 3–14-flowered	cyme 12–23-flowered	cyme 3–25-flowered
Leaf blade	spatulate, rarely obovate or broadly obovate, apex obtuse, base cuneate, densely glandular	spatulate, apex acute, base obscurely spurred, sparsely pubescent	triangular to broadly triangular-ovate, apex acute, base broadly cuneate to truncate, densely glandular
Leaf dimensions (length × width)	5–20 × 3.5–13 mm	7–9 × 2.2–3 mm	7–15 × 7–9 mm
Sepal	obovate or broadly obovate, apex obtuse, base shortly spurred, densely glandular	lanceolate to spatulate, apex acute, basal spur inconspicuous, sparsely pubescent	lanceolate to oblong, apex acuminate, densely glandular
Seed size (length × width)	ca. 0.56 × 0.32 mm	ca. 0.29 × 0.15 mm	ca. 0.38 × 0.24 mm

### Pollen and seed micromorphology

**Figure 1.**
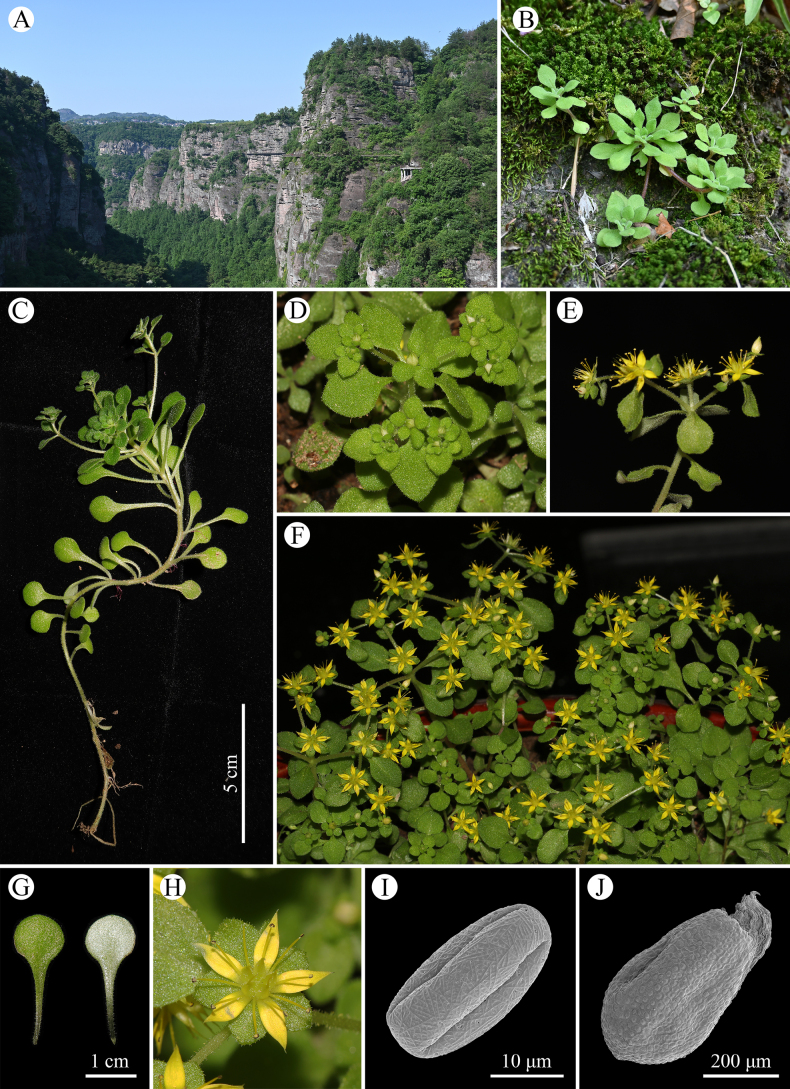
Photos of *Sedum xinchangense* sp. nov. **A**. Habitat; **B**. Habit; **C**. Sterile plant; **D**. Plant before flowering; **E**. Flowering branch; **F**. Habit (cultivated); **G**. Adaxial and abaxial leaf; **H**. Flower; **I**. Pollen grain; **J**. Seed.

Scanning electron microscopy revealed that the pollen grains of the new species are tricolporate and prolate to perprolate in shape, with a perforate-reticulate tectal ornamentation (Fig. 1). The pollen measures 26.14 ± 1.77 µm in polar axis and 12.74 ± 0.69 µm in equatorial diameter, yielding a polar/equatorial (P/E) ratio of 2.05 ± 0.13. The seeds are ovoid and display a reticulate-unipapillate surface pattern characterized by straight anticlinal walls. Seed dimensions are 0.56 ± 0.04 mm in length and 0.32 ± 0.04 mm in width, with a length-to-width ratio of 1.77 ± 0.14.

### Phylogenetic relationships

The lengths of the four aligned sequences are 676 bp (ITS), 1058 bp (*matK*), 812 bp (*rps16*), and 377 bp (*trnL-F*). Under the BIC, the optimal nucleotide substitution models selected were SYM+I+G for ITS, TVM+G for *matK*, and TPM1uf+G for both *rps16* and *trnL-F*.

**Figure 2.**
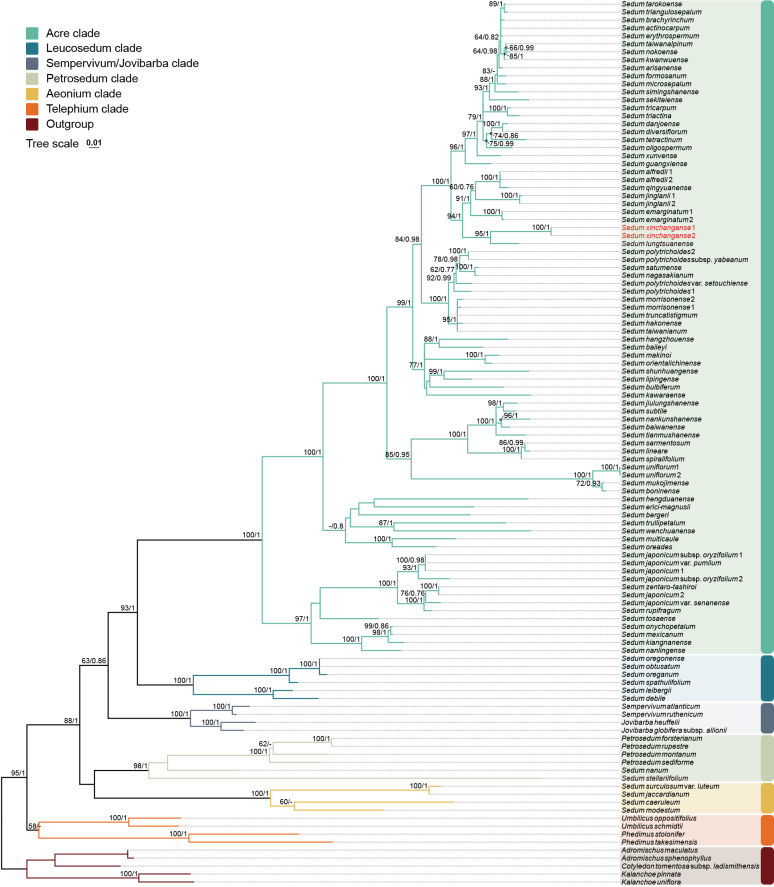
Maximum Likelihood phylogenetic tree, based on ITS sequences. The numbers above the branches are bootstrap values (BS) and Bayesian Posterior Probabilities (PP). ‘-’ indicates BS < 50% or PP < 0.75. The new species is highlighted in red.

Phylogenetic analyses of ITS dataset using ML and BI yielded largely congruent topologies (Fig. 2, Suppl. material 2: fig. S1). The ITS ML phylogenetic tree places the new species, *S. xinchangense*, within the *Acre* clade of subfamily Sempervivoideae. The two accessions of the new species formed a strongly supported clade (BS = 100%, PP = 1) and were resolved as sister to *S. lungtsuanense* with strong support (BS = 95%, PP = 1). In contrast, *S. stellariifolium* was placed in the *Petrosedum* clade of the same subfamily.

Phylogenetic trees based on three plastid genes (*matK*, *rps16*, and *trnL-F*) also showed that the two samples of the new species formed a strongly supported clade (BS = 100%, PP = 1) (Suppl. material 3: fig. S2, Suppl. material 4: fig. S3). However, as sequence data for the morphologically similar *S. lungtsuanense* and *S. stellariifolium* were unavailable, their phylogenetic positions relative to the new species could not be assessed using plastid markers. Resolution within the plastid trees was generally poor: the ML tree showed low bootstrap support at most nodes, while the BI tree exhibited polytomies at multiple nodes, suggesting limited phylogenetic signal in these plastid markers.

## Conclusion

The ITS phylogenetic tree shows that the new species belongs to the *Acre* clade within the subfamily Sempervivoideae (Fig. 2). The two samples of the new species, *S. xinchangense*, formed a strongly supported clade (BS = 100%, PP = 1), and demonstrate a sister relationship with *S. lungtsuanense* (Fig. 2, Suppl. material 2: fig. S1). At the same time, the new species forms a highly supported clade with sect. *Sedum* members *S. lungtsuanense*, *S. emarginatum*, *S. jinglanii*, *S. qingyuanense*, and *S. alfredii* ( BS = 94%, PP = 1).

Morphologically, the new species resembles *S. lungtsuanense* and *S. stellariifolium* but can be distinguished by a suite of characters, including stolons, leaf and sepal shape, indumentum type, flower number, and leaf and seed size (see Diagnosis and Table 1 for details). Biologically, *S. xinchangense* is a perennial herb occurring in dry, exposed cliff habitats, whereas *S. lungtsuanense* is annual and grows in shaded, moist environments, and *S. stellariifolium* is annual or biennial and occupies both dry and moist habitats.

Based on morphological characters and phylogenetic relationships, *S. xinchangense* is confirmed as a distinct new species, and we propose placing it in sect. *Sedum*.

## Taxonomic treatment

### Sedum xinchangense

Taxon classificationPlantaeSaxifragalesCrassulaceae

Z.D.Wang, X.F.Jin & Y.F.Lu sp. nov.

A0792BB7-B13E-5AD3-AB66-66B607BB9885

urn:lsid:ipni.org:names:77378389-1

Figs 1, 3

#### Chinese name.

xīn chāng jǐng tiān (新昌景天).

**Figure 3.**
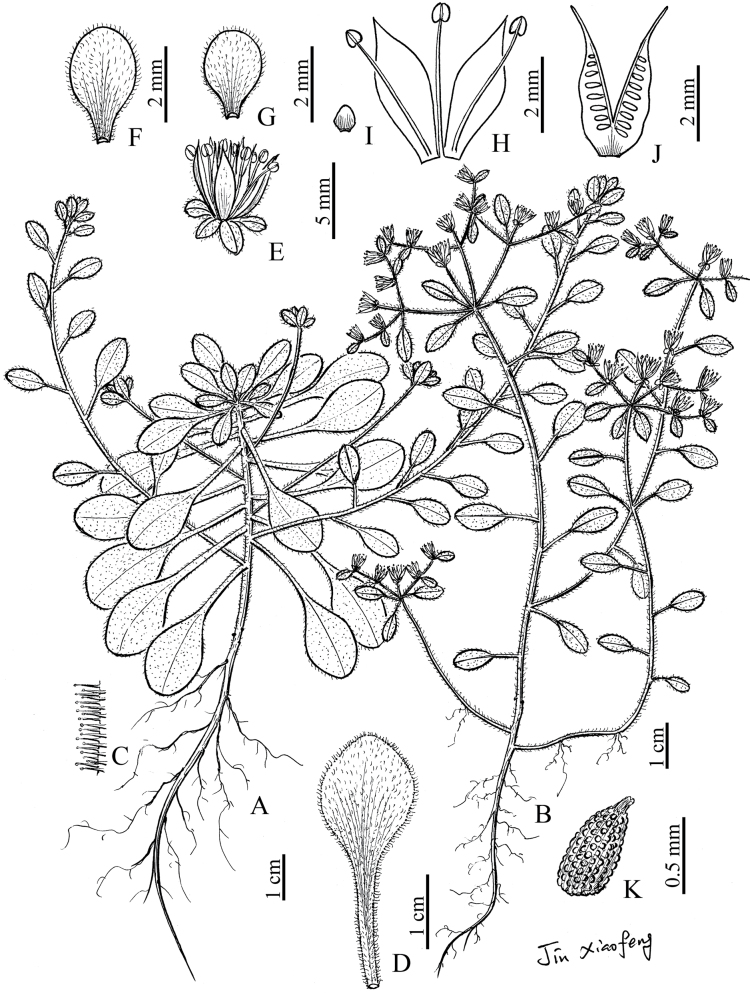
*Sedum xinchangense* sp. nov. **A, B**. Habit; **C**. Glandular hair; **D**. Leave; **E**. Flower; **F, G**. Sepals; **H**. Petals and stamens; **I**. Nectar scale; **J**. Opened unripe follicles; **K**. Seed (drawn by Xiao-Feng Jin from the holotype).

#### Diagnosis.

*Sedum xinchangense* is morphologically similar to *S. lungtsuanense* and *S. stellariifolium* but differs by its basal stolons (absent in the latter two), densely glandular stems, leaves, and bracts (vs. sparsely pubescent in *S. lungtsuanense*), 3–14-flowered cymes (vs. 12–23-flowered in *S. lungtsuanense* and 3–25-flowered in *S. stellariifolium*), spatulate leaf blade, rarely obovate or broadly obovate, (vs. triangular to broadly triangular-ovate in *S. stellariifolium*), and obovate or broadly obovate sepals (vs. lanceolate to spatulate in *S. lungtsuanense* and lanceolate to oblong in *S. stellariifolium*). Additionally, it has consistently larger leaves and seeds: leaves 5–20 × 3.5–13 mm (vs. 7–9 × 2.2–3 mm in *S. lungtsuanense* and 7–15 × 7–9 mm in *S. stellariifolium*), seeds ca. 0.56 × 0.32 mm (vs. ca. 0.29 × 0.15 mm in *S. lungtsuanense* and ca. 0.38 × 0.24 mm in *S. stellariifolium*).

#### Type.

China • Zhejiang Province: Hangzhou City, Lin’an District, cultivated in Zhejiang A&F University, introduced from Mt. Shijiufeng of Xinchang County, 18 Jun. 2025, *Z. D. Wang 20250618* (holotype: ZJFC!; isotypes: ZM!).

#### Description.

Herbs perennial. Roots fibrous, slender. Stems ascending, both sterile stem and flowering stem with stolons at base, succulent, 7–15 cm tall, with dense white spreading glandular hairs; basal nodes with fibrous adventitious roots. Leaves on sterile stems alternate, upper ones densely aggregated, those on stolons frequently alternate, nearly verticillate at the top, and those on flowering stems alternate, sometimes 3-verticillate at the top; leaf blades succulent, spatulate, rarely obovate or broadly obovate, 5–20 mm long, 3.5–13 mm wide, apex obtuse, base cuneate, densely glandular on both surfaces, with costa and lateral veins inconspicuous; petioles 3–18 mm long, densely glandular, base shortly spurred. Cyme 3- or 2-branched, scorpioid-spreading, 3–14-flowered; bracts frondose, ovate or ovate-round, 2.5–9 mm long, 2–8 mm wide, apex obtuse, base cuneate and short-stipitate, both surfaces densely glandular. Flowers bisexual, center ones pedunculate, others subsessile. Sepals 5, obovate or broadly obovate, 2–3 mm long, 1–1.5 mm wide, apex obtuse, base shortly spurred, both surfaces densely glandular. Petals 5, yellow, oblong-ovate, slightly longer than sepals, 3.5–4.5 mm long, ca. 1.5 mm wide, apex acuminate, sometimes outside sparsely glandular. Stamens 10; antisepalous ones ca. 3.5 mm long; antipetalous ones ca. 3 mm long, inserted nearly from petal base; anthers ovoid, orange to purple-black. Nectar scales 5, triangle-ovate or broadly spatulate, minute, apex obtuse. Carpels 5, erect, oblong-lanceolate, 4–4.5 mm long, connate for ca. 1 mm long at base, sometimes sparsely pubescent; styles ca. 1 mm long, slender. Follicles divergent, many-seeded. Seeds minute, pale brown, ovoid, ca. 0.5 mm long, ca. 0.3 mm wide.

#### Etymology.

The specific epithet ‘xinchangense’ refers to the type locality of this new species.

#### Phenology.

Flowering and fruiting from June to July.

#### Distribution and habitat.

The new species is only known from Mt. Shijiufeng of Xinchang County, Zhejiang. It is currently known to grow on cliffs at lower elevations of 200–220 m.

#### Additional specimen examined

**(paratypes)**. China • Zhejiang Province: Xinchang County, Mt. Shijiufeng, 29.37333628°N, 120.81258862°E, on cliffs, elev. 210 m, 12 May 2025, *Z. D. Wang & Q. G. Li 2025051201* (ZJFC, ZM), *2025051202* (ZJFC).

#### Conservation status.

*Sedum xinchangense* is assessed as Vulnerable (VU) according to the IUCN Red List (IUCN 2024). The species is currently known only from Mt. Shijiufeng, Xinchang County, Zhejiang Province, China. It occurs on cliffs adjacent to tourist trails and comprises about 12 populations with fewer than 1,000 mature individuals in total. Although the population appears stable at present, it is directly threatened by ongoing and increasing tourist activities, which cause habitat disturbance through trail maintenance, trampling, and potential rock face modifications. Under IUCN criteria D1 and D2, based on its fewer than 1,000 mature individuals and its highly restricted distribution with susceptibility to future anthropogenic disturbance, the species meets the criteria for classification as VU.

#### Notes.

The micromorphology of testa in Crassulaceae can be categorized into four principal types based on two key characters: the morphology of the anticlinal walls (sinuate vs. straight) and the number of papillae per cell (one, two, or multiple). These are: (1) sinuate-unipapillate, (2) straight-unipapillate, (3) straight-bipapillate, and (4) straight-multipapillate (‘t Hart and Berendsen 1980; Knapp 1994; Thiede and Eggli 2007). The reticulate-unipapillate form of the new species corresponds to the characteristic seed sculpture of the Acre clade, in which lateral cell walls are thickened into a distinct reticulate pattern, typically bearing a central papilla. This micromorphological correspondence not only supports the placement of the new species within the *Acre* clade but also underscores the taxonomic utility of seed-coat sculpture as a stable diagnostic feature for this lineage.

In a separate SEM-based survey of seed morphology across 26 *Sedum* s.l. species from Zhejiang, Jin et al. (2008) recognized five seed types based on size, shape, and surface sculpture: *Orostachys*-type, *Hylotelephium*-type, *Phedimus*-type, *Filipes*-type, and *Sedum*-type. The seed micromorphology of the new species is consistent with that of the *Sedum*-type, supporting its systematic position within the sect. *Sedum*.

##### Key to the species of Sedum from Zhejiang, East China

**Table:** 

1a	Flowers pedicellate at base, without bracts	**2a**
2a	Annual plants; flowers white-pink or pale purple	**1. *S. drymarioides***
2b	Annual or biennial plants; flowers yellow	**2. *S. stellariifolium***
1b	Flowers sessile or subsessile, each with a bract at base	**3a**
3a	Plants with fertile and sterile stems; leaves dimorphic on fertile stems and sterile stems	**4a**
4a	Leaves on sterile stems 1.2–2.5 cm long, 0.7–1.8 cm wide, emarginate at apex	**5a**
5a	Leaves 3- or 4-verticillate on sterile stems	**3. *S. kiangnanense***
5b	Leaves alternate on sterile stems	**4. *S. tosaense* subsp. *sinense***
4b	Leaves on sterile stems 0.8–2 cm long, 0.5–1 cm wide, obtuse at apex	**6a**
6a	Leaves 3- or 4-verticillate on sterile stems	**5. *S. subtile***
6b	Leaves alternate on sterile stems	**6. *S. formosanum***
3b	Leaves monomorphic on fertile stems and sterile stems, or plants with only fertile stems, sometimes leaves dimorphic on fertile stems	**7a**
7a	Leaves dimorphic on fertile stems	**8a**
8a	Flower solitary; leaves alternate	**7. *S. hoi***
8b	Flowers more than 10 in a cyme; lower leaves 4-verticillate	**8. *S. zhenghaianum***
7b	Leaves monomorphic on fertile stems and sterile stems	**9a**
9a	Stems, leaves and bracts all glandular or pubescent	**10a**
10a	Plants with stolons at base; stems, leaves and bracts densely glandular; cymes 3–14-flowered	**9. *S. xinchangense* sp. nov**.
10b	Plants without stolons; stems, leaves and bracts sparsely pubescent; cymes 12–23-flowered	**10. *S. lungtsuanense***
9b	Stems, leaves and bracts glabrous	**11a**
11a	Leaves alternate on fertile stems	**12a**
12a	Leaves and bracts ovate to obovate-round	**13a**
13a	Petals and pistils/carples 5	**11. *S. oligospermum***
13b	Petals and pistils/carples 4	**14a**
14a	Leaves attenuate at base, subsessile	**12. *S. dongzhiense***
14b	Leaves long-petiolate at base	**13. *S. tetractinum***
12b	Leaves and bracts linear to spatulate	**15a**
15a	Plants woody at base; leaves densely alternate, moss-like	**14. *S. polytrichoides***
15b	Plants herbaceous; leaves sparsely alternate, deciduous	**16a**
16a	Plants tuberous; bracts longer than flowers	**15. *S. leptophyllum***
16b	Plants without tubers; bracts shorter to slightly longer than flowers	**17a**
17a	Plants with bulbils at upper leaf axils	**16. *S. bulbiferum***
17b	Plants without bulbils	**18a**
18a	Plants < 15 cm tall; leaves linear to lanceolate	**19a**
19a	Plants < 10 cm tall, not branched	**17. *S. tianmushanense***
19b	Plants 8–15 cm tall, branched at base	**18. *S. japonicum***
18b	Plants > 15 cm tall; leaves spatulate-oblong to spatulate-obovate	**20a**
20a	Pistils/carples 5	**21a**
21a	Plants branched at base; anthers orange-red	**22a**
22a	Sepals broadly linear to triangular, nearly equal in length	**19. *S. hangzhouense***
22b	Sepals spatulate to obovate, unequal	**20. *S. xunvense***
21b	Plants branched at base to middle; anthers yellow or purple-brown	**23a**
23a	Plants branched at base; leaves terete	**21. *S. yongkangense***
23b	Plants branched at middle; leaves flat, spatulate or spatulate-obovate	**24a**
24a	Biennial plants; anthers yellow	**22. *S. simingshanense***
24b	Perennial plants; anthers purple-brown	**23. *S. alfredii***
20b	Pistils/carples 3, rarely 2 or 4	**24. *S. tricarpum***
11b	Leaves opposite or verticillate on fertile stems	**25a**
25a	Plants erect or nearly erect; leaves opposite, rarely 3-verticillate	**26a**
26a	Leaves emarginate at apex	**25. *S. emarginatum***
26b	Leaves obtuse at apex	**27a**
27a	Plants not branched, erect	**26. *S. baileyi***
27b	Plants branched, nearly erect	**28a**
28a	Sepals 2–4 mm long; petals 3–5 mm long	**29a**
29a	Cymes 2-branched; sepals spatulate-obelliptic	**27. *S. orientalichinense***
29b	Cymes 2–4-branched; sepals linear-spatulate	**30a**
30a	Sepals linear-spatulate; leaves 6–8 mm wide	**28. *S. makinoi***
30b	Sepals linear; leaves ca. 2.5 mm wide	**29. *S. erythrospermum***
28b	Sepals 5–9 mm long; petals 7–8 mm long	**30. *S. kuntsunianum***
25b	Plants erect or repens; leaves verticillate	**31a**
31a	Plants erect; leaves 3- or 4-verticillate; leaves on sterile stems linear	**32a**
32a	Leaves 10–15 mm long; seeds densely papillate	**31. *S. lineare***
32b	Leaves 5–10 mm long; seeds sparsely papillate	**32. *S. onychopetalum***
31b	Plants repens; leaves 3-verticillate; leaves on sterile stems ovovate, oblanceolate ot oblong	**33a**
33a	Stems slender; leaves obovate	**33. *S. jiulungshanense***
33b	Stems thick; leaves oblanceolate or oblong	**34. *S. sarmentosum***

## Supplementary Material

XML Treatment for Sedum xinchangense

## Additional information

### Use of AI

No use of AI was reported.

### Author ORCIDs

Zhi-Dan Wang https://orcid.org/0009-0001-2588-0775

Xiao-Feng Jin https://orcid.org/0000-0002-8195-6738

Yi-Fei Lu https://orcid.org/0000-0001-6095-3821

### Data availability

All of the data that support the findings of this study are available in the main text or Supplementary Information.

## Supplementary materials

Supplementary material 1 GenBank accession number of species from NCBI Data type pdf https://binary.pensoft.net/file/1585833This dataset is made available under the Open Database License (http://opendatacommons.org/licenses/odbl/1.0/). The Open Database License (ODbL) is a license agreement intended to allow users to freely share, modify, and use this Dataset while maintaining this same freedom for others, provided that the original source and author(s) are credited. Zhi-Dan Wang, Xiao-Feng Jin, Yi-Fei Lu

Supplementary material 2 Bayesian phylogenetic tree based on ITS sequences. The new species is highlighted in red, and morphologically similar species are highlighted in blue Data type pdf https://binary.pensoft.net/file/1585834This dataset is made available under the Open Database License (http://opendatacommons.org/licenses/odbl/1.0/). The Open Database License (ODbL) is a license agreement intended to allow users to freely share, modify, and use this Dataset while maintaining this same freedom for others, provided that the original source and author(s) are credited. Zhi-Dan Wang, Xiao-Feng Jin, Yi-Fei Lu

Supplementary material 3 Maximum Likelihood phylogenetic tree based on three plastid genes (*matK*, *rps16*, and *trnL-F)* Data type pdf Explanation note Phylogenetic tree based on three plastid genes (*matK*, *rps16*, and *trnL-F*). https://binary.pensoft.net/file/1585835This dataset is made available under the Open Database License (http://opendatacommons.org/licenses/odbl/1.0/). The Open Database License (ODbL) is a license agreement intended to allow users to freely share, modify, and use this Dataset while maintaining this same freedom for others, provided that the original source and author(s) are credited. Zhi-Dan Wang, Xiao-Feng Jin, Yi-Fei Lu

Supplementary material 4 Bayesian phylogenetic tree based on three plastid genes (*matK*, *rps16*, and *trnL-F*) Data type pdf https://binary.pensoft.net/file/1585836This dataset is made available under the Open Database License (http://opendatacommons.org/licenses/odbl/1.0/). The Open Database License (ODbL) is a license agreement intended to allow users to freely share, modify, and use this Dataset while maintaining this same freedom for others, provided that the original source and author(s) are credited. Zhi-Dan Wang, Xiao-Feng Jin, Yi-Fei Lu

## References

[B1] Andrews S (2019) FastQC a quality control tool for high throughput sequence data. Babraham bioinformatics. https://www.bioinformatics.babraham.ac.uk/projects/fastqc/

[B2] APG IV (2016) An update of the Angiosperm Phylogeny Group classification for the orders and families of flowering plants: APG IV. Botanical Journal of the Linnean Society 181: 1–20. 10.1111/boj.12385

[B3] Carrillo-Reyes P, Sosa V, Mort ME (2009) Molecular phylogeny of the *Acre* clade (Crassulaceae): Dealing with the lack of definitions for *Echeveria* and *Sedum*. Molecular Phylogenetics and Evolution 53: 267–276. 10.1016/j.ympev.2009.05.022 19482091

[B4] Chai ML, Wu YM, Yang Z, Pu JB, Chen B, Xu YL, Li P (2024) *Sedum xunvense*, a new species from Southeast China. Phytotaxa 644(4): 258–270. 10.11646/phytotaxa.644.4.2

[B5] Chang H, Zhang L, Xie H, Liu J, Xi Z, Xu X (2021) The conservation of chloroplast genome structure and improved resolution of infrafamilial relationships of Crassulaceae. Frontiers in Plant Science 12: e631884. 10.3389/fpls.2021.631884 PMC8281817 34276716

[B6] Chen Y, Chen Y, Shi C, Huang Z, Zhang Y, Li S, Li Y, Ye J, Yu C, Li Z, Zhang X, Wang J, Yang H, Fang L, Chen Q (2018) SOAPnuke: a MapReduce acceleration-supported software for integrated quality control and preprocessing of high-throughput sequencing data. GigaScience 7(1): 1–6. 10.1093/gigascience/gix120 PMC5788068 29220494

[B7] Collins TJ (2007) ImageJ for microscopy. Biotechniques 43(1 Suppl): 25–30. 10.2144/000112517 17936939

[B8] Dai JM, Xiong Y, Li P, Xu YL, Fan Q (2025) A new species of *Sedum* (Crassulaceae) from eastern China based on morphological and molecular evidence. PhytoKeys 253: 271–285. 10.3897/phytokeys.253.119922 PMC11929005 40123974

[B9] Darriba D, Taboada GL, Doallo R, Posada D (2012) jModelTest 2: more models, new heuristics and parallel computing. Nature Methods 9(8): 772–772. 10.1038/nmeth.2109 PMC4594756 22847109

[B10] Ding H, Han S, Ye Y, Bi D, Zhang S, Yi R, Gao J, Yang J, Wu L, Kan X (2022) Ten plastomes of *Crassula* (Crassulaceae) and phylogenetic implications. Biology 11(12): e1779. 10.3390/biology11121779 PMC9775174 36552287

[B11] Doyle JJ, Doyle JL (1987) A rapid DNA isolation procedure for small quantities of fresh leaf tissue. Phytochemical Bulletin 19: 11–15.

[B12] Forbes FB, Hemsley WB (1887) An enumeration of all the plants known from China Proper, Formosa, Hainan, Corea, the Luchu Archipelago, and the Island of Hongkong, together with their distribution and synonymy. Journal of the Linnean Society of London, Botany 23(155): e286. 10.1111/j.1095-8339.1887.tb00532.x

[B13] Fu KJ, Rao GY (1988) New taxa of subgenus *Sedum* and a new combination of *Hylotelephium* from China. Acta Botanica Boreali-Occidentalia Sinica 8(2): 116–124.

[B14] Fu KT, Ohba H (2001) Crassulaceae. In: Wu ZY, Raven PH (Eds) Flora of China (Vol. 8). Science Press, Beijing, China and Missouri Botanical Garden Press, St. Louis, USA, 202–268.

[B15] Fu SH (1965) Species et Combinationes Novae Crassulacearum Sinicarum. Acta Phytotax Sinica Additamentum 1: e115.

[B16] Fu SH, Fu KT (1984) *Sedum*. In: Fu SH, Fu KT (Eds) Flora Reipublicae Popularis Sinicae. Science Press, Beijing, China 34(1): 31–220.

[B17] Gontcharova SB, Gontcharov AA (2009) Molecular phylogeny and systematics of flowering plants of the family Crassulaceae DC. Molecular Biology 43: 794–803. 10.1134/s0026893309050112 19899633

[B18] Guindon S, Gascuel O (2003) A simple, fast, and accurate algorithm to estimate large phylogenies by maximum likelihood. Systematic Biology 52(5): 696–704. 10.1080/10635150390235520 14530136

[B19] Han S, Zhang S, Yi R, Bi D, Ding H, Yang J, Ye Y, Xu W, Wu L, Zhuo R, Kan X (2024) Phylogenomics and plastomics offer new evolutionary perspectives on Kalanchoideae (Crassulaceae). Annals of Botany 133(4): 585–604. 10.1093/aob/mcae017 PMC11037489 38359907

[B20] ‘t Hart H, Berendsen W (1980) Ornamentation of the testa in *Sedum* (Crassulaceae). Plant Systematics and Evolution 135: 107–117. 10.1007/BF00983011

[B21] Ho YQ (1989) New taxa of *Sedum* from Zhejiang. Bulletin of Botanical Research 9(4): 31–35.

[B22] Hsu PS (1991) Contributions to the flora of southeastern China II. Rheedea 1(1&2): 44–51.

[B23] Hu BZ, Huang XL, Shu P (1981) Studies on the species of *Sedum sarmentosum* and *S. angustifolium*. Acta Phytotax Sinica 19(3): 308–312.

[B24] IUCN (2024) Guidelines for using the IUCN Red List categories and criteria, version 16. Prepared by the Standards and Petitions Committee. https://www.iucnredlist.org/resources/redlistguidelines

[B25] Jin SH, Zhou YY, Ding BY, Wang RW, Jin XF (2013) *Sedum kuntsunianum* (Crassulaceae: Sedoideae), a new species from southern Zhejiang, China. Phytotaxa 105(2): 33–38. 10.11646/phytotaxa.105.2.1

[B26] Jin XF, Qian L, Lu YH, Zhang HW, Wang HZ (2008) Seed micromorphology of *Sedum* (s.l.) from Zhejiang and its taxonomic implications. Journal of Zhejiang University (Agric. & Life Sci.) 34(4): 409–417. 10.3785/j.issn.1008-9209.2008.04.010

[B27] Jin XF, Zhang HW, Xie JB, Zhao YJ (2010) Taxonomic notes on *Sedum* s. str. (Crassulaceae) from Zhejiang Province,China. Journal of Hangzhou Normal University (Natural Science Edition) 9(3): 165–171+190.

[B28] Knapp U (1994) Skulptur der samenschale und gliederung der Crassulaceae. Botanische Jahrbücher für Systematik, Pflanzengeschichte und Pflanzengeographie 116: 157–187.

[B29] Kumar S, Stecher G, Tamura K (2016) MEGA7: Molecular evolutionary genetics analysis version 7.0 for bigger datasets. Molecular Biology and Evolution 33(7): 1870–1874. 10.1093/molbev/msw054 PMC8210823 27004904

[B30] Liu XY, Zhang DQ, Zhang JQ (2023) Plastomic data shed new light on the phylogeny, biogeography, and character evolution of the family Crassulaceae. Journal of Systematics and Evolution 61(6): 990–1003. 10.1111/jse.13003

[B31] Messerschmid TFE, Klein JT, Kadereit G, Kadereit JW (2020) Linnaeus’s folly–phylogeny, evolution and classification of *Sedum* (Crassulaceae) and Crassulaceae subfamily Sempervivoideae. Taxon 69(5): 892–926. 10.1002/tax.12316

[B32] Migo H (1937) Note on the flora of south-eastern China III. Journal of the Shanghai Science Institute 3: e224.

[B33] Mort ME, O’Leary TR, Carrillo-Reyes P, Nowell T, Archibald JK, Randle CP (2010) Phylogeny and evolution of Crassulaceae: past, present, and future. Biodiversity Ecology 3: 69–86.

[B34] Mort ME, Soltis DE, Soltis PS, Francisco‐Ortega J, Santos‐Guerra A (2001) Phylogenetic relationships and evolution of Crassulaceae inferred from *matK* sequence data. American Journal of Botany 88(1): 76–91. 10.2307/2657129 11159129

[B35] Ronquist F, Teslenko M, van der Mark P, Ayres DL, Darling A, Höhna S, Larget B, Liu L, Suchard MA, Huelsenbeck JP (2012) MrBayes 3.2: efficient Bayesian phylogenetic inference and model choice across a large model space. Systematic Biology 61(3): 539–542. 10.1093/sysbio/sys029 PMC3329765 22357727

[B36] She SQ, Zhang Y, Zhou X, Peng YJ, Yao SH, Zhao XX, Yang J, Xu YL (2025) *Sedum simingshanense* (Crassulaceae), a new species from Zhejiang, East China. PhytoKeys 251: 23–35. 10.3897/phytokeys.251.125595 PMC11742097 39829712

[B37] Stamatakis A (2014) RAxML version 8: a tool for phylogenetic analysis and post-analysis of large phylogenies. Bioinformatics 30(9): 1312–1313. 10.1093/bioinformatics/btu033 PMC3998144 24451623

[B38] Stephenson R (1994) *Sedum*: Cultivated Stonecrops. Timber Press, Portland, 355 pp.

[B39] Thiede J, Eggli U (2007) Crassulaceae. In: Kubitzki K (Ed.) Flowering Plants Eudicots. The Families and Genera of Vascular Plants (Vol. 9). Springer, Berlin/Heidelberg, 83–118. 10.1007/978-3-540-32219-1_12

[B40] Van Ham R, Hart H (1998) Phylogenetic relationships in the Crassulaceae inferred from chloroplast DNA restriction-site variation. American Journal of Botany 85: 123–134. 10.2307/2446561 21684886

[B41] Wai CM, Weise SE, Ozersky P, Mockler TC, Michael TP, VanBuren R (2019) Time of day and network reprogramming during drought induced CAM photosynthesis in *Sedum album*. PLoS Genet 15(6): e1008209. 10.1371/journal.pgen.1008209 PMC6594660 31199791

[B42] Wang H, Song XJ, Liu QW (2005) *Sedum hoi*, a new species of the Crassulaceae from Zhejiang, China. Acta Botanica Yunnanica 27(3): 381–382.

[B43] Wu LH, Liu YJ, Guo FG, Bi D, Guo XH, Baker AJM, Smith JAC, Luo YM (2013) *Sedum plumbizincicola* X.H. Guo et S.B. Zhou ex L.H. Wu (Crassulaceae): a new species from Zhejiang Province, China. Plant Systematics and Evolution 299: 487–498. 10.1007/s00606-012-0738-x

[B44] Xie JM, Chen YR, Cai GJ, Cai RL, Hu Z, Wang H (2023) Tree visualization by one table (tvBOT): a web application for visualizing, modifying and annotating phylogenetic trees. Nucleic Acids Research 51(W1): W587–W592. 10.1093/nar/gkad359 PMC10320113 37144476

[B45] Xie PL, Guo YL, Teng Y, Zhou WB, Yu Y (2024) GeneMiner: a tool for extracting phylogenetic markers from next-generation sequencing data. Molecular Ecology Resources 24(3): e13924. 10.1111/1755-0998.13924 38197287

[B46] Zhang D, Gao FL, Jakovlić I, Zou H, Zhang J, Li WX, Wang GT (2020) PhyloSuite: an integrated and scalable desktop platform for streamlined molecular sequence data management and evolutionary phylogenetics studies. Molecular Ecology Resources 20(1): 348–355. 10.1111/1755-0998.13096 31599058

