# Supplementary material for: Sedum xinchangense, a new species of Crassulaceae from Zhejiang, East China
Source: PhytoKeys. 2026 Apr 7;272:181–96. doi: 10.3897/phytokeys.272.188016 (PMC13080381; doi:10.3897/phytokeys.272.188016)
Supplement: Supplementary material 1 — GenBank accession number of species from NCBI [file phytokeys-272-181_article-188016__-s001.pdf]

Table S1. List of species from the Crassulaceae subfamilies Sempervivoideae and Kalanchoideae sampled in this study, with GenBank accession numbers for the nuclear ribosomal internal transcribed spacer (ITS) and three plastid regions (*matK*, *rps16*, and *trnL-trnF*).

Taxon	ITS	matK	rps16	trnL-F
**Acre clade**				
Sedum actinocarpum	LC229264	LC258185	LC229446	LC229514
Sedum alfredii 1	AB930260	-	LC229431	LC229499
Sedum alfredii 2	AB930261	LC258165	LC229432	LC229500
Sedum arisanense	LC229272	LC258187	LC229453	LC229521
Sedum baileyi	PP981035	FJ919945	-	-
Sedum baiwanense	PV476760	-	-	-
Sedum bergeri	AY352897	-	-	-
Sedum boninense	LC229242	-	-	-
Sedum brachyrinchum	LC229275	LC258192	LC229458	LC229526
Sedum bulbiferum	LC229234	AF115652	LC229434	JQ954574
Sedum danjoense	LC260127	-	LC860756	LC860766
Sedum diversiflorum	LC860733	-	-	-
Sedum emarginatum 1	KM111145	-	-	KM111121
Sedum emarginatum 2	PP981038	-	-	-
Sedum erici magnusii	LC229235	-	-	-
Sedum erythrospermum	AB906473	-	-	AB932631
Sedum formosanum	AB930272	LC258195	LC229462	LC229530
Sedum guangxiense	OL693034	-	-	-
Sedum hakonense	AB930278	LC258168	LC229435	LC229503
Sedum hangzhouense	LC260130	-	-	-
Sedum hengduanense	ON707679	-	-	-
Sedum japonicum 1	AB906475	KX452274	-	AB089784
Sedum japonicum 2	LC229237	LC618899	-	-
Sedum japonicum subsp. *oryzifolium* 1	LC229239	AF115647	-	AB089786
Sedum japonicum subsp. *oryzifolium* 2	AB088618	-	-	-
Sedum japonicum var. *pumilum*	LC229240	-	-	-
Sedum japonicum var. *senanense*	LC229238	-	-	-
Sedum jinglanii 1	OP288035	-	-	-
Sedum jinglanii 2	OQ162326	-	-	-
Sedum jiulungshanense	LC229243	-	-	-
Sedum kawaraense	LC731693	-	-	-
Sedum kiangnanense	LC229244	-	-	-
Sedum kwanwuense	LC229293	-	LC229485	LC229553

Sedum lineare	AB088623	-	-	AB089773
Sedum lipingense	MN150061	-	-	MN434078
Sedum lungtsuanense	LC260131	-	-	-
Sedum makinoi	AB906476	LC258169	LC229436	AB089779
Sedum mexicanum	LC229247	-	-	AB089783
Sedum microsepalum	LC229282	LC258207	-	-
Sedum morrisonense 1	LC229290	AF115651	-	AB932630
Sedum morrisonense 2	LC229289	-	LC229484	LC229552
Sedum mukojimense	LC530823	-	-	-
Sedum multicaule	AB088631	-	-	AB089782
Sedum nagasakianum	LC229249	LC258170	LC229437	LC229505
Sedum nankunshanense	PV476757	-	-	-
Sedum nanlingense	MN105949	-	-	-
Sedum nokoense	LC229294	LC258219	LC229486	LC229554
Sedum oligospermum	LC229250	MN492869	LC860755	LC860765
Sedum onychopetalum	KM111148	-	-	KM111124
Sedum oreades	KF113733	-	-	KF113839
Sedum orientalichinense	PP116144	-	-	-
Sedum polytrichoides 1	KM111143	LC258172	-	-
Sedum polytrichoides 2	LC229252	-	LC229438	LC229506
Sedum polytrichoides subsp. *yabeanum*	AB906490	-	LC229439	AB932634
Sedum polytrichoides var. *setouchiense*	LC229253	-	LC229440	LC229508
Sedum qingyuanense	PX560092	-	-	-
Sedum rupifragum	LC229254	-	-	-
Sedum sarmentosum	LC229255	AF115649	-	AB089774
Sedum satumense	LC229256	LC258174	LC229441	LC229509
Sedum sekiteiense	LC229295	LC258220	LC229487	LC229555
Sedum shunhuangense	PV589377	-	-	-
Sedum simingshanense	PP464048	-	-	-
Sedum spiralifolium	KM111160	-	-	KM111138
Sedum subtile	AB088622	KX452273	-	AB089775
Sedum taiwanalpinum	LC229278	-	LC229459	LC229527
Sedum taiwanianum	LC229297	LC258222	LC229489	LC229557
Sedum tarokoense	LC229298	LC258223	LC229490	LC229558
Sedum tetractinum	LC260135	-	LC860757	LC860767
Sedum tianmushanense	LC229261	-	-	-
Sedum tosaense	AB088620	-	-	AB089787
Sedum triactina	AB088629	-	-	AB089780
Sedum triangulosepalum	LC229299	LC258224	LC229491	LC229559
Sedum tricarpum	LC229259	LC258176	LC229442	LC229510
Sedum trullipetalum	AB088630	-	-	AB089781

Sedum truncatistigmum	LC229306	-	LC229498	LC229565
Sedum uniflorum 1	LC229241	-	-	-
Sedum uniflorum 2	LC530832	-	-	-
Sedum wenchuanense	ON707681	-	-	-
Sedum xunvense	PV101948	-	-	-
Sedum zentaro-tashiroi	AB088619	-	-	AB089785
**Leucosedum clade**				
Sedum debile	HE999651	KX452265	-	-
Sedum leibergii	MT336117	-	-	-
Sedum obtusatum	MT336129	-	-	-
Sedum oreganum	HE999673	KX452264	-	-
Sedum oregonense	KJ884128	MT181588	KJ884337	MT155917
Sedum spathulifolium	MT336136	-	-	MT155920
**Sempervivum/Jovibarba clade**				
Jovibarba globifera subsp. *allionii*	KJ884135	-	KJ884341	MT155886
Jovibarba heuffelii	KJ884152	MT181558	KJ884359	MT155887
Sempervivum atlanticum	KJ884188	MT181597	KJ884385	MT155927
Sempervivum ruthenicum	KJ884292	-	KJ884483	-
**Petrosedum clade**				
Petrosedum forsterianum	KJ884125	MT181574	KJ884334	MT155903
Petrosedum rupestre	MT336134	AF115667	KJ884338	-
Petrosedum montanum	HE999667	-	-	-
Petrosedum sediforme	HE999683	AF115640	-	X74286
Sedum nanum	MT336126	MT181586	-	MT155915
Sedum stellariifolium	MH711568	-	-	-
**Aeonium clade**				
Sedum caeruleum	MT336105	MT181573	OM337425	MT155902
Sedum jaccardianum	AY082100	AF115637	-	AY082220
Sedum modestum	AY082101	AF115639	OM337433	AY082221
Sedum surculosum var. *luteum*	LM993288	KX452239	OM337430	OM337466
**Telephium clade**				
Phedimus stolonifer	MT336091	MT181559	-	MT155888
Phedimus takesimensis	JQ954566	-	-	-
Umbilicus oppositifolius	KJ884131	MT181598	-	MT155928
Umbilicus schmidtii	KP279434	KP279376	-	KP279334
**Outgroups**				
Adromischus maculatus	AY692317	AF115575	-	AY692287
Adromischus sphenophyllus	AY692318	-	-	AY692288
Cotyledon tomentosa subsp. *ladismithensis*	AY692311	AF115592	-	AY692302

Kalanchoe pinnata	AJ231323	GU135118	-	-
Kalanchoe uniflora	AJ231343	AF115623	-	-
