# Supplementary figures and images for: Sedum xinchangense, a new species of Crassulaceae from Zhejiang, East China
Source: PhytoKeys. 2026 Apr 7;272:181–96. doi: 10.3897/phytokeys.272.188016 (PMC13080381; doi:10.3897/phytokeys.272.188016)

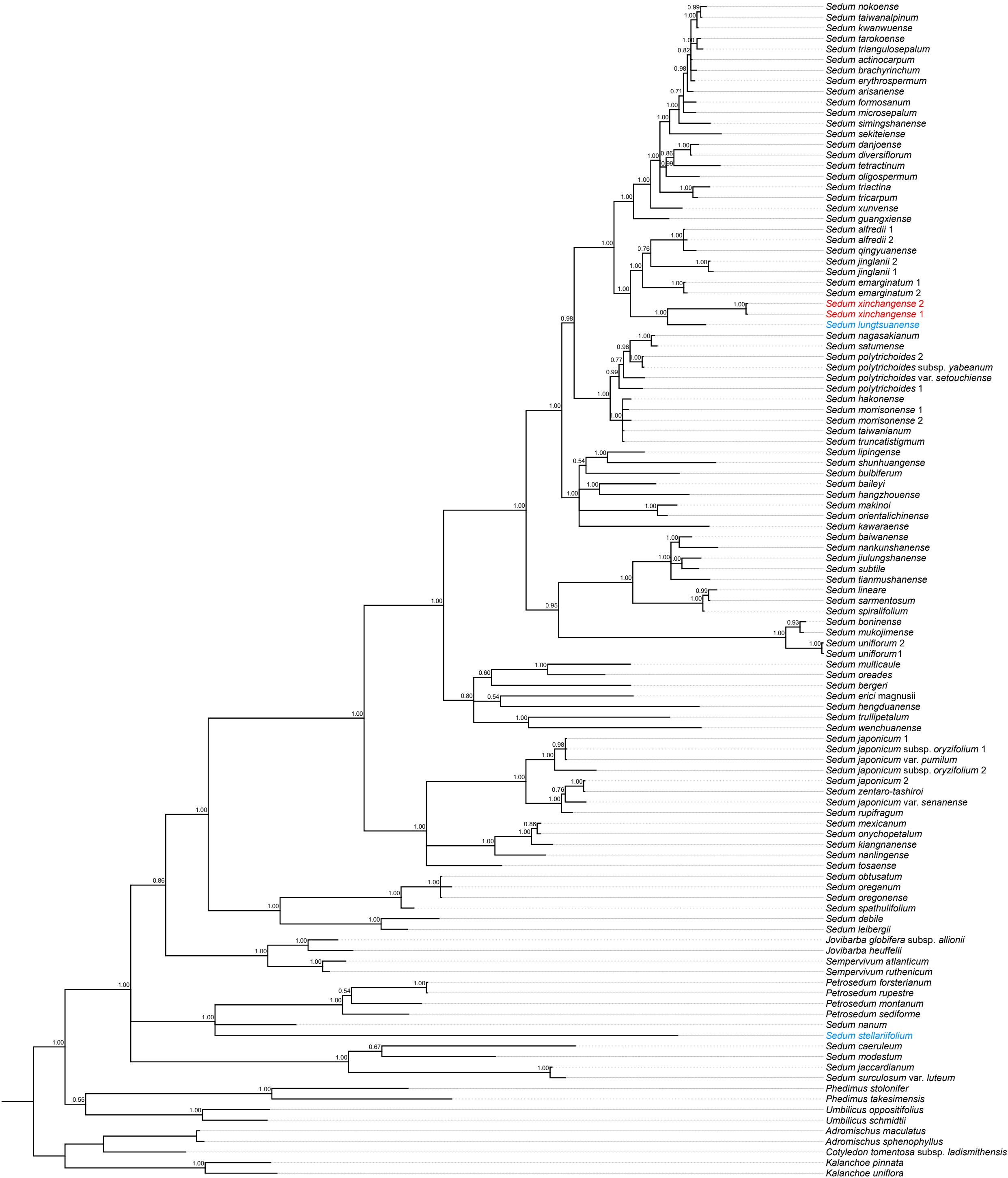

Supplement: Supplementary material 2 — Bayesian phylogenetic tree based on ITS sequences. The new species is highlighted in red, and morphologically similar species are highlighted in blue [file phytokeys-272-181_article-188016__-s002.pdf]

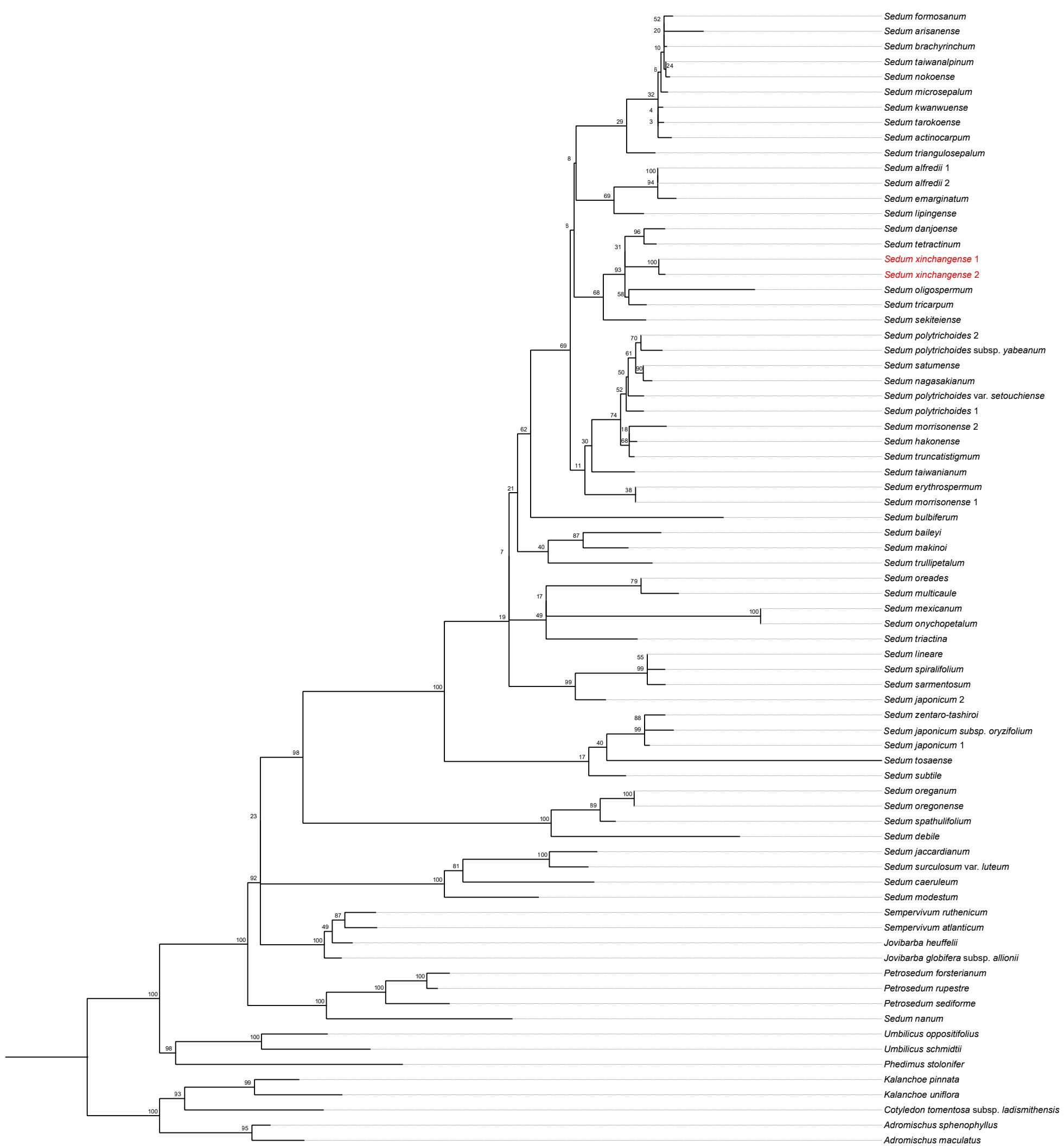

Supplement: Supplementary material 3 — Maximum Likelihood phylogenetic tree based on three plastid genes (matK, rps16, and trnL-F) [file phytokeys-272-181_article-188016__-s003.pdf]

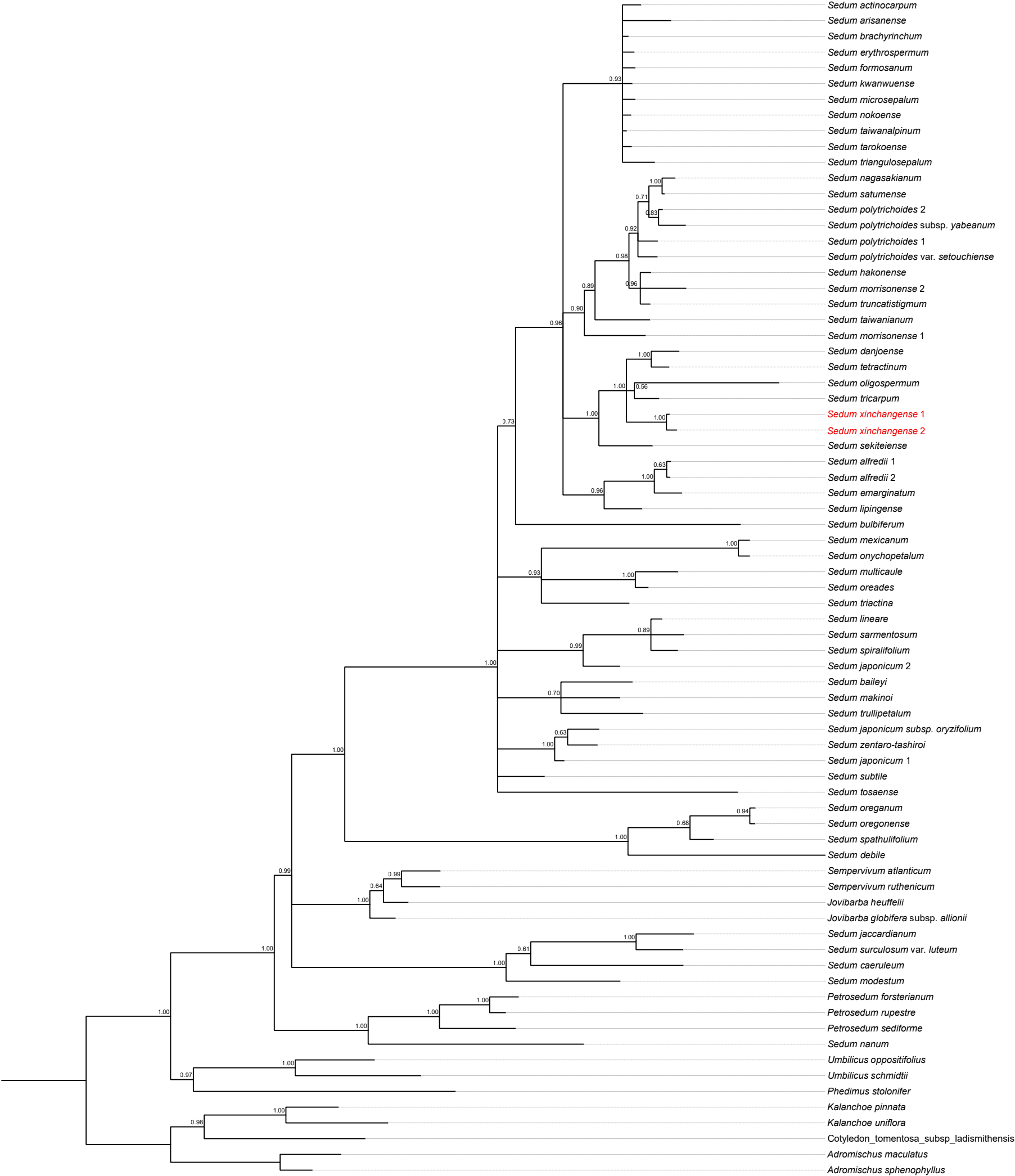

Supplement: Supplementary material 4 — Bayesian phylogenetic tree based on three plastid genes (matK, rps16, and trnL-F) [file phytokeys-272-181_article-188016__-s004.pdf]
